# Photochemical Reactivity of Humic Substances in an Aquatic System Revealed by Excitation-Emission Matrix Fluorescence

**DOI:** 10.3389/fchem.2021.679286

**Published:** 2021-05-28

**Authors:** Xin-yuan Wang, Qi-peng Yang, Shi-jie Tian, Fan-hao Song, Fei Guo, Nan-nan Huang, Wei-qiang Tan, Ying-chen Bai

**Affiliations:** ^1^ School of Environmental and Municipal Engineering, Qingdao University of Technology, Qingdao, China; ^2^ State Key Laboratory of Environmental Criteria and Risk Assessment, Chinese Research Academy of Environmental Sciences, Beijing, China

**Keywords:** humic substance, photochemical reactivity, excitation-emission matrix fluorescence, fluorescence regional integration, parallel factor analysis, kinetic model

## Abstract

The photochemical reactivity of humic substances plays a critical role in the global carbon cycle, and influences the toxicity, mobility, and bioavailability of contaminants by altering their molecular structure and the mineralization of organic carbon to CO_2_. Here, we examined the simulated irradiation process of Chinese standard fulvic acid (FA) and humic acid (HA) by using excitation-emission matrix fluorescence combined with fluorescence regional integration (FRI), parallel factor (PARAFAC) analysis, and kinetic models. Humic-like and fulvic-like materials were the main materials (constituting more than 90%) of both FA and HA, according to the FRI analysis. Four components were identified by the PARAFAC analysis: fulvic-like components composed of both carboxylic-like and phenolic-like chromophores (C1), terrestrial humic-like components primarily composed of carboxylic-like chromophores (C2), microbial humic-like overwhelming composed of phenolic-like fluorophores (C3), and protein-like components (C4). After irradiation for 72 h, the maximum fluorescence intensity (*F*_max_) of C1 and C2 of FA was reduced to 36.01–58.34%, while the *F*_max_ of C3 of both FA and HA also decreased to 0–9.63%. By contrast, for HA, the *F*_max_ of its C1 and C2 increased to 236.18–294.77% when irradiated for 72 h due to greater aromaticity and photorefractive tendencies. The first-order kinetic model (*R* ^2^ = 0.908–0.990) fitted better than zero-order kinetic model (*R* ^2^ = 0–0.754) for the C1, C2, and C3, of both FA and HA, during their photochemical reactivity. The photodegradation rate constant (*k*_1_) of C1 had values (0.105 for FA; 0.154 for HA) that surpassed those of C2 (0.059 for FA, 0.079 for HA) and C3 (0.079 for both FA and HA) based on the first-order kinetic model. The half-life times of C1, C2, and C3 ranged from 6.61–11.77 h to 4.50–8.81 h for FA and HA, respectively. Combining an excitation-emission matrix with FRI and PARAFAC analyses is a powerful approach for elucidating changes to humic substances during their irradiation, which is helpful for predicting the environmental toxicity of contaminants in natural ecosystems.

## Introduction

Humic substances (HSs) are mixture of heterogeneous organic molecules that are ubiquitous in terrestrial and aquatic ecosystems, playing an essential role in biogeochemical and ecological processes (Du et al., 2016). HSs can be categorized as fulvic acid (FA), humic acid (HA), and humin according to their degree of water solubility (Bai et al., 2015). HSs are significant portion of aquatic dissolved organic matter (DOM), which are isolated and purified by ruling out hydrophilic acid, lipids, proteins etc. (Kitis et al., 2001). The photochemical reactivity of DOM produces dissolved inorganic carbon (Gao, 1998), in addition to organic molecules of low molecular weight (Kulovaara, 1996; Gonsior et al., 2014) and complex aromatic structures (Timko et al., 2015), all of which could influence the toxicity, mobility, and transformation of aquatic contaminants (Kida et al., 2019). Many previous studies have focused on the photodegradation of non-purified DOM in natural water (Zhu et al., 2017; Kida et al., 2019). Recently, the photochemical reactivity of DOM samples varying in molecular mass from Yangtze River and its coastal area were examined by excitation-emission matrix (EEM) spectroscopy, which revealed that the highly aromatic humic-like materials exhibited greater photochemical reactivity than did the non-humic-like materials (Zhu et al., 2017). Photodegradation was confirmed to be the major external factor driving the compositional diversity of DOM in Antarctic lakes and streams, as assessed using the spectral slope S_275–279_ for the distribution of its fluorescence components (Kida et al., 2019). Nonetheless, how photochemical reactivity variously acts upon the structures and functions of HSs is still not well understood; hence, the proper isolation and purification of HSs is critical to elucidate photochemical reactivity mechanisms.

Fluorescence regional integration (FRI), a powerful method to determine the volume integral under each region, has been widely utilized to characterize the composition spatiotemporal changes of DOM from various environments, including rivers, wastewaters, and soils (Chen et al., 2003; Xie et al., 2017; Wang et al., 2019). For example, when used with EEM, FRI was successful at distinguishing the decrease of protein-like materials and increase of humic-like materials across the wastewater treatment plant–river–lake continuum (Wang et al., 2019). Also, EEM coupled with a parallel factor (PARAFAC) analysis has been widely used to study the photodegradation of DOM in different aquatic systems (Ishii and Boyer, 2012; Du et al., 2016; Retelletti Brogi et al., 2020). For example, this EEM-PARAFAC approach was able to reveal the effect of light on PARAFAC components by exposing the DOM from a sub-alpine lake to three different light sources (Du et al., 2016). Seven PARAFAC components were identified by (Kida et al., 2019), who studied the photodegradation of DOM from 47 lakes and two streams on the ice-free area at Lützow-Holm Bay and Amundsen Bay in East Antarctica. Five primary components in the Yangtze River and two primary components in the Western Pacific Ocean were identified by PARAFAC analysis during irradiation of DOM measured by EEM, the relative abundance of photo-refractory UVC humic-like components was increased during irradiation (Zhu et al., 2017). But the possible applications of using EEM in tandem with both FRI and PRAFAC to characterize the variation in HSs during irradiation have yet to widely reported on.

First-order and zero-order kinetic models are powerful ways of describing the photochemical reactivity of DOM, or of organic contaminants in the presence of DOM (Filipe et al., 2020). For instance, the first-order kinetic was used to describe the photodegradation dynamics of DOM in the Lake Biwa watershed in Japan and during two characteristic seasons in the Negro River (Rodríguez-Zúñiga et al., 2008; Mostofa et al., 2010), and also applied to leaf litter-derived humic substances after irradiation for 0–12 days (Hur et al., 2011). The first-order kinetic was also used to model the photodegradation of DOM with TiO_2_ as a catalyst in stormwater runoff (Zhao et al., 2018). The zero-kinetic model was used describe the degradation of propranolol by photo-Fenton in the presence of humic substances (López-Vinent et al., 2020). However, such suitable kinetic models have not been adequately studied to further explore the photodegradation of FA and HA.

Accordingly, this study aimed to 1) investigate the variation in EEM of both FA and HA during irradiation; 2) distinguish the changed PARAFAC components in the photochemical reactivity process, and; 3) determine the kinetic model that best describes the photochemical reactivity dynamics of FA and HA.

## Materials and Methods

### Samples Pretreatment and Characterization

Soil samples were collected from Jiufeng Mountain in Beijing, China. Both FA and HA were isolated and purified by the XAD-8 resin adsorption technique according to the International Humic Substances Society (IHSS, http://humic-substances.org). Detailed information was previously reported by Bai et al. (2015) on the collection and isolation of Chinese Standard FA and HA. A certain amount of sieved of soil was dissolved by water, the FA and HA were separated from the soil after a series of acidification, alkalization and centrifuge at high speed. The FA solution was adsorbed on XAD-8 resin column, and the FA sample was obtained by freeze drying after elution with NaOH solution, rinsing with water, removal impurity by HF and purification with H+-saturated cation exchange resin. Redissolved the HA fraction by adding KOH under N_2_, add solid KCl and centrifuge at high speed to remove the suspended solids. Reprecipitate the humic acid by adding HCl with constant stirring and allow the suspension to stand again. Centrifuge and discard the supernatant. Suspend the HA precipitate in HF solution and shake. Centrifuge and repeat the HCl/HF treatment, until the ash content is below 1%. Transfer the precipitate to a Visking dialysis tube and dialyze against distilled water, freeze dry the HA. The data of UV-Vis, NMR, and elemental compositions for FA and HA was provided in the supporting information (Supplementary Figure S1; Supplementary Tables S1–S3). All the chemicals used here were of analytical reagent grade, unless otherwise mentioned. All the solutions were prepared in Milli-Q water and filtered through a 0.45-µm glass fiber membrane (Whatman, United Kingdom).

### Photochemical Experiments

To carry out the photochemical reaction of HSs, photochemical reactor equipped with a 1000 W xenon lamp was used to provide the simulated radiation of the solar spectrum. Its quartz tubes were soaked in 10% HNO_3_ for 12 h before rinsing them with Milli-Q water and then drying them at 100°C for 2 h prior to their use. Light intensity was measured to be 0.78 mW/cm^2^, at 290–420 nm, on the surface of the quartz tubes. The schematic diagram of the photochemical reactor was shown in Supplementary Figure S2

Both FA and HA were prepared separately at a concentration of 10.0 mg/L in a 0.02 mol/L NaOH solution, to effectively dissolve each. The ionic strength of FA and HA solutions were adjusted to 0.01 M NaCl (Wang et al., 2015). The pH of each solution was adjusted to 6.0 ± 0.02, by injecting minimal amounts of HCl or NaOH as needed, with 15 min allowed for equilibration. Next, 50 ml of these HSs’ solutions were transferred to cleaned quartz tubes, these then sealed with Teflon caps to minimize possible evaporation and absorption. During the irradiation experiments, photochemical reactor’s temperature was maintained at 25°C by circulated cooling water. The samples of HSs were determined after illumination for 0, 2, 4, 8, 12, 22, 32, 52, and 72 h, respectively with fluorescence spectroscopy. All the experiments were carried out in duplicate including UV illumination and determination with fluorescence spectroscopy.

### Fluorescence Measurement

The EEM spectra of the HSs samples were recorded on a Hitachi F-7000 fluorescence spectrometer, using quartz cuvettes with a 1-cm path length, at room temperature. Its scanning emission (Em) wavelength spanned 250–550 nm (increments of 2 nm) and excitation (Ex) wavelength ranged from 200 to 400 nm (increments of 2 nm). For FA, the Ex and Em slit widths were both 5 nm but for HA they were 5 and 10 nm, respectively. The scanning speed was set to 2,400 nm/min and the photomultiplier voltage was set to 600 V. The EEM of 0.1 M NaCl blank was subtracted from EEMs of FA and HA. The Rayleigh scattering of FA and HA with time was shown in Supplementary Figure S3. Rayleigh scattering values did not change significantly with increasing irradiation time.

### FRI Analysis

FRI as a quantitative analysis technique used to divide the EEM into five regions (i.e., Region I–V) (Chen et al., 2003; Song et al., 2018). Here, FRI parameters of fluorescence responses ($P_{\text{i}\text{n}}\%$) were calculated using this equation (Song et al., 2018):

$$P_{\text{i}\text{n}}=\frac{{Ф}_{\text{i}\text{n}}}{{Ф}_{\text{T}\text{n}}}\;\times \;100\%=\frac{MF_{\text{i}}{\sum^{\text{​}}}_{\text{ex}}{\sum^{\text{​}}}_{\text{em}}I\left(\lambda_{\text{ex}}\lambda_{\text{em}}\right)\Delta \lambda_{\text{ex}}\Delta \lambda_{\text{em}}}{\sum_{i=1}^{5}{Ф}_{\text{i}\text{n}}}\;\times \;100\%$$ (1)

where ${Ф}_{\text{i}\text{n}}$ and ${Ф}_{\text{T}\text{n}}$ are the Ex/Em area volumes corresponding to that of region i (i = I–V) and the total region, respectively; the $MF_{\text{i}}$ is a multiplication factor for each region; $I\left(\lambda_{\text{ex}}\lambda_{\text{em}}\right)$ is the fluorescence intensity at the excitation wavelength and emission wavelength; $\Delta \lambda_{\text{ex}}$ and $\Delta \lambda_{\text{em}}$ are the increments of excitation wavelength and emission wavelength, respectively. The FRI analysis was implemented in MATLAB 2009 and the boxplots of $P_{\text{i}\text{n}}$ were drawn in Origin software 2018.

### PARAFAC Analysis

Through PARAFAC modeling, the three-way data of an EEM can be statistically reduced to trilinear terms and a residual array, expressed as follows:

$$X_{ijh\;}=\;\overset{N}{\underset{n=1}{\sum }}a_{if}b_{jf}c_{hf}\;+\;\varepsilon_{ijh}\;i\;=\;1Ij\;=\;1Jh\;=\;1H$$ (2)

where $X_{ijh}$ is the fluorescence intensity of the sample i at the Em wavelength j and Ex wavelength h; N is the number of components; $a_{if}$ is directly proportional to the concentration of the fluorescence component f in sample i; $b_{jf}$ and $c_{hf}$ are respective estimates of Em and Ex spectra for the fluorescence component f; $\varepsilon_{ijh}$ is the residual term, which is the variance unexplained by the model (He et al., 2013; Song et al., 2017).

A total of 36 EEMs of FA and HA were used for the PARAFAC analysis by the DOMFluor toolbox (version 1.7) in MATLAB software. Importantly, the EEMs of the NaCl blank were subtracted from each EEMs of FA and HA, to eliminate any water Raman scattering peaks from arising, with a series of zero values inserted to the region of no fluorescence (i.e., Ex << Em) to minimize the effects of scattering lines of EEMs. Any residual Rayleigh and Raman scattering that appeared was regulated by the interpolation method derived from Bahram et al. (2006), Song et al. (2017). The 2–7 components model of PARAFAC was applied to the EEMs of both FA and HA. The split-half analysis, residual analysis, and visual inspection were all used to determine the correct number of final components (Stedmon and Bro, 2008; Wu et al., 2011; Zhang et al., 2020). The maximum fluorescence intensity (*F*_max_) derived by the PARAFAC model represented the relative intensity or concentration of the PARAFAC components (Guo et al., 2015; Maqbool and Hur, 2016). The model loadings obtained from the PARAFAC analysis were uploaded into OpenFluor (https://openfluor.lablicate.com), in which a query was conducted to match the spectral properties from our loadings with existing data available from other studies.

### Apparent Kinetic Model of Photochemical Reactivity

To establish the changes of HSs during irradiation, the apparent kinetic models were used to research the photochemical reactivity of HSs. The possible rate equation of photochemical reactivity of HSs follows the zero-order kinetic model (*n* = 0) and the first-order kinetic model (*n* = 1), whose equations are expressed as follows:

$$F\;=\;F_{0}-k_{0}t$$ (3)

$$ln\left(\frac{F\;\;-\;F_{end}}{F_{0}\;-\;F_{end}}\right)\;=\;-\;k_{1}t$$ (4)

where $F_{0}$ and F are the initial fluorescence values (the *F*_0_ baseline defined as 100 units) and fluorescence with irradiation, respectively; $F_{end}$ is the final fluorescence intensity caused by irradiation; $k_{0}$ and $k_{1}$ are the respective rate constants of the zero-order kinetic and first-order kinetic model. The $T_{12}^{0}$ and $T_{12}^{1}$ terms are the half-life values of zero-order and first-order kinetic models, given by:

$$T_{12}^{0}\;=\;\frac{F\;\;-\;F_{0}}{k_{0}}$$ (5)

$$T_{12}^{1}\;=\;\frac{ln2}{k_{1}}$$ (6)

All parameter values of *k*, $T_{12}$, $F_{end}$, and *R* ^2^ were estimated in SigmaPlot software (Co., United States).

## Result and Discussion

### General EEMs of HSs

**FIGURE 1:**
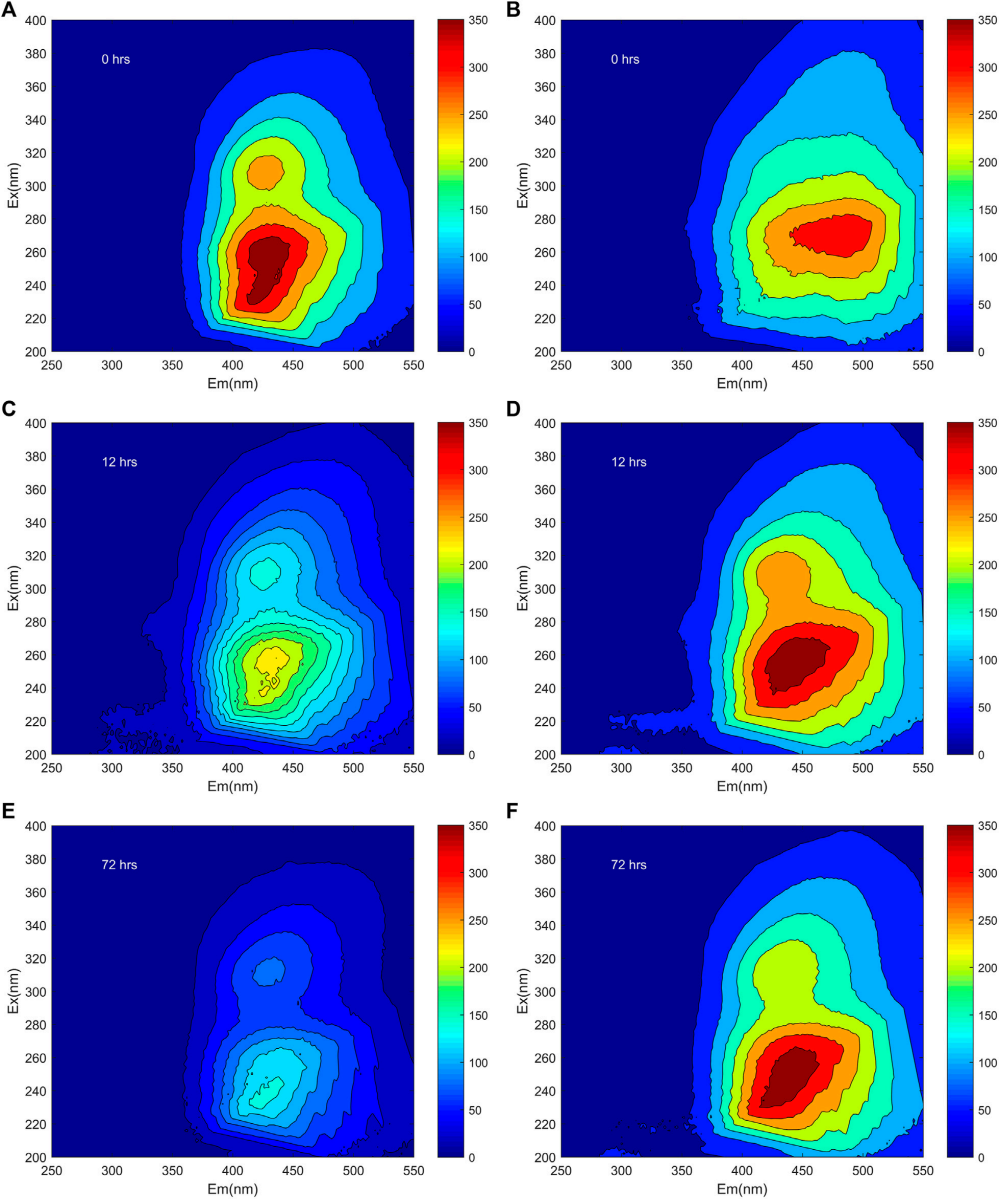
The EEMs of both FA **(A,C,E)** and HA **(B,D,F)** at pH 6 after their irradiation for 0, 12, and 72 h, respectively.

Before the photochemical reaction, the general EEMs exhibited three main peaks for FA, these named Peak A (Ex/Em: 308–312/422–434 nm), Peak B (Ex/Em: 254–260/430–446 nm), Peak C (Ex/Em: 228–234/424–444 nm), respectively (Figure 1; Supplementary Table S1). One main peak for HA was located at Ex/Em: 272/484 nm, here named Peak D, before the irradiation (Figure 1; Supplementary Table S1). According to previous research, Peak A, Peak B, and Peak D are attributable to humic-like materials, while Peak C corresponds to fulvic-like materials (Sun et al., 2016; Song et al., 2017). Specifically, the Em of Peak D of HA was longer than that of Peak B of FA (Figure 1, Supplementary Table S1). A longer Ex/Em of soil humic acid may indicate greater aromaticity and a larger scale of the π-electron system (Yu et al., 2020). This longer Ex/Em of HA could also arise from it harboring a higher amount of conjugated aromatic π-electron systems with electron-withdrawing functional groups when compared with FA.

The fluorescence intensities both of Peak A, B, and C for FA decreased during irradiation. In detail, the fluorescence intensity of Peak A, Peak B, and Peak C decreased to 54.6, 61.2, and 59.6% rapidly during irradiation of 0–12 h, respectively, and the fluorescence intensity of Peak A and Peak C decreased to 31.6 and 43.3% gradually during irradiation of 12–72 h, while, the Peak B of FA was disappeared at the end of irradiation (Figure 1; Supplementary Table S1). Declines in fluorescence intensity during irradiation were also obtained during the photodegradation of DOM in Lake Baihua and Lake Hongfeng (Zhang et al., 2020). Interestingly, during the irradiation the Peak D of HA split into Peak A, B, and C. Photolysis of humic substances in this study was firstly considered to be carried out by direct photolysis, which involves energy and electron transfer after absorption of photons by humic substances (Xu et al., 2011). In the study of photodegradation of Suwannee River fulvic acid and eutrophic lake-derived DOM, it was suggested that the reduction in fluorescence intensity can be explained by the removal of aromatic chromophores and/or the degradation of aromatic compounds to produce humic substances photoproducts that do not absorb UV light (Hur et al., 2011; Xu and Jiang, 2013). Previous research has shown that photodegradation of DOM in sub-alpine lake resulted in increase in fluorescence intensity, the increase of fluorescent substances may be due to some aromatic chromophores with resistance to photodegradation or the formation of aromatic humic substances compounds during irradiation experiments (Du et al., 2016).

The positions of peaks changed in varying degrees, for both FA and HA, across the irradiation time 0–72 h. The clear red shifts of Em for Peak C in FA were observed at 6 and 8 nm after irradiation for 12 and 72 h, respectively (Figure 1A; Supplementary Table S1). After irradiation, a red shift was also found for the Ex/Em of Peak A, the Em of Peak B, and the Em of Peak C for HA (Figure 1B; Supplementary Table S1). Red shifts were also reported during the irradiation of DOM in Lake Hongfeng and colloidal organic matter in Yangtze River (Zhu et al., 2017; Zhang et al., 2020), in those cases being related to more carbonyl, hydroxyl, alkoxy, and amino groups in the DOM molecules (Yu et al., 2020). The slight red shifts for both FA and HA likely arose from possible changes in conformation that occurred during irradiation with simulating solar energy.

### FRI Analysis of HSs

FRI was utilized here to quantitatively analyze the EEM spectra and identify different fluorescence materials, with EEM of the HSs then classified into five regions (Regions I–V) according to the previous reports (Chen et al., 2003). Both Region I (Ex/Em: 200–250/250–330 nm) and Region II (Ex/Em: 200–250/330–380 nm) were related to simple aromatic proteins, namely tyrosine-like and tryptophan-like materials, respectively. Additionally, Region III (Ex/Em: 200–250/380–550 nm), Region IV (Ex/Em: 250–450/250–380 nm), and Region V (Ex/Em: 250–450/380–550 nm) were respectively classified as fulvic-like, soluble microbial byproduct-like, and humic-like materials (Chen et al., 2003; Song et al., 2018). Peak A (Ex/Em: 308–312/428–434 nm), Peak B (Ex/Em: 254–260/432–446 nm), and Peak D (Ex/Em: 272/484 nm) were all located in Region V, thus corresponding to humic-like materials, while Peak C (Ex/Em: 227–237/424–444 nm), located in Region III, corresponded to fulvic-like materials.

**FIGURE 2:**
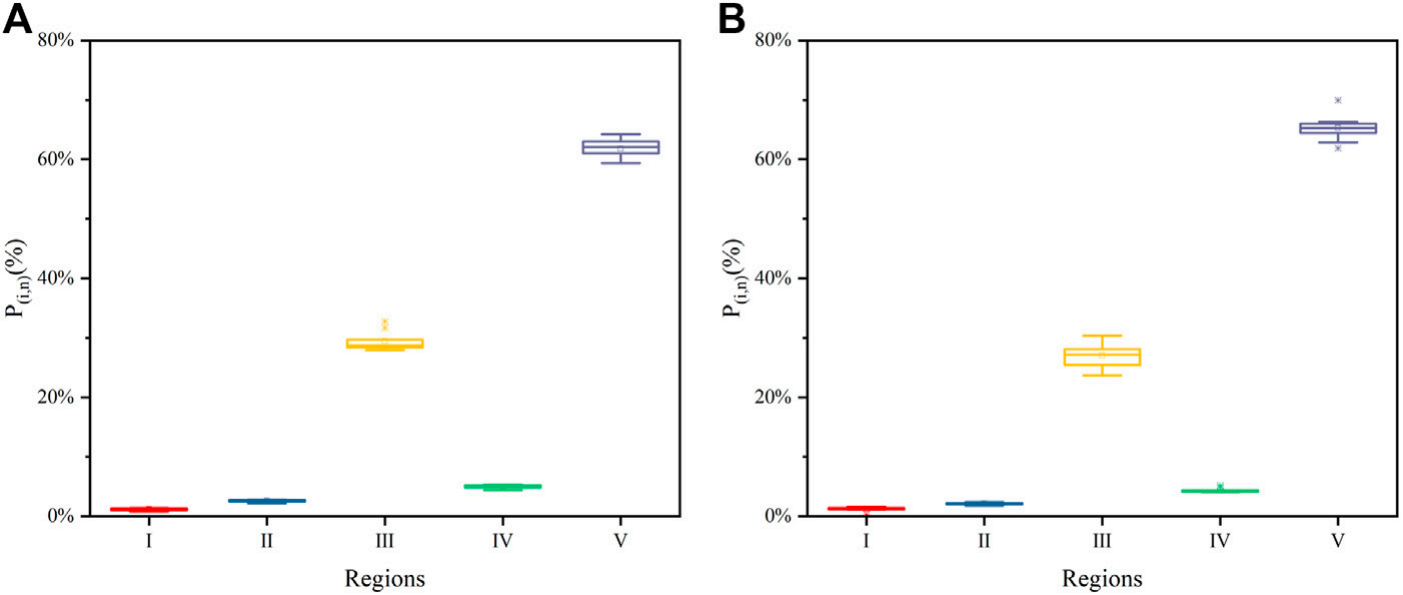
Boxplots of fluorescence response parameter’s (*P*_i,n_) distributions in the EEMs regions of both FA **(A)** and HA **(B)**

Figure 2 shows the regional distributions of FA and HA as boxplots. For FA, the *P*_i,n_ of regions I–V had values of 1.16 ± 0.19%, 2.59 ± 0.18%, 29.44 ± 1.57%, 5.01 ± 0.28%, and 61.81 ± 1.54%, respectively (Figure 2A), while those of HA were 1.22 ± 0.35%, 2.12 ± 0.17%, 27.00 ± 2.08%, 4.40 ± 0.35%, and 65.26 ± 2.15%, respectively (Figure 2B). The sum of *P*_III,n_ and *P*_V,n_ was more than 90%, indicating that fulvic-like and humic-like were the main materials of FA and HA. Mean *P*_i,n_ and its interquartile range were used to compare the contents of specific materials in the HSs (Wei et al., 2016). In this way, the contents of fluorescent materials were ranked as humic-like > fulvic-like > soluble microbial byproduct-like > tryptophan-like > tyrosine-like materials, for both FA and HA.

**FIGURE 3:**
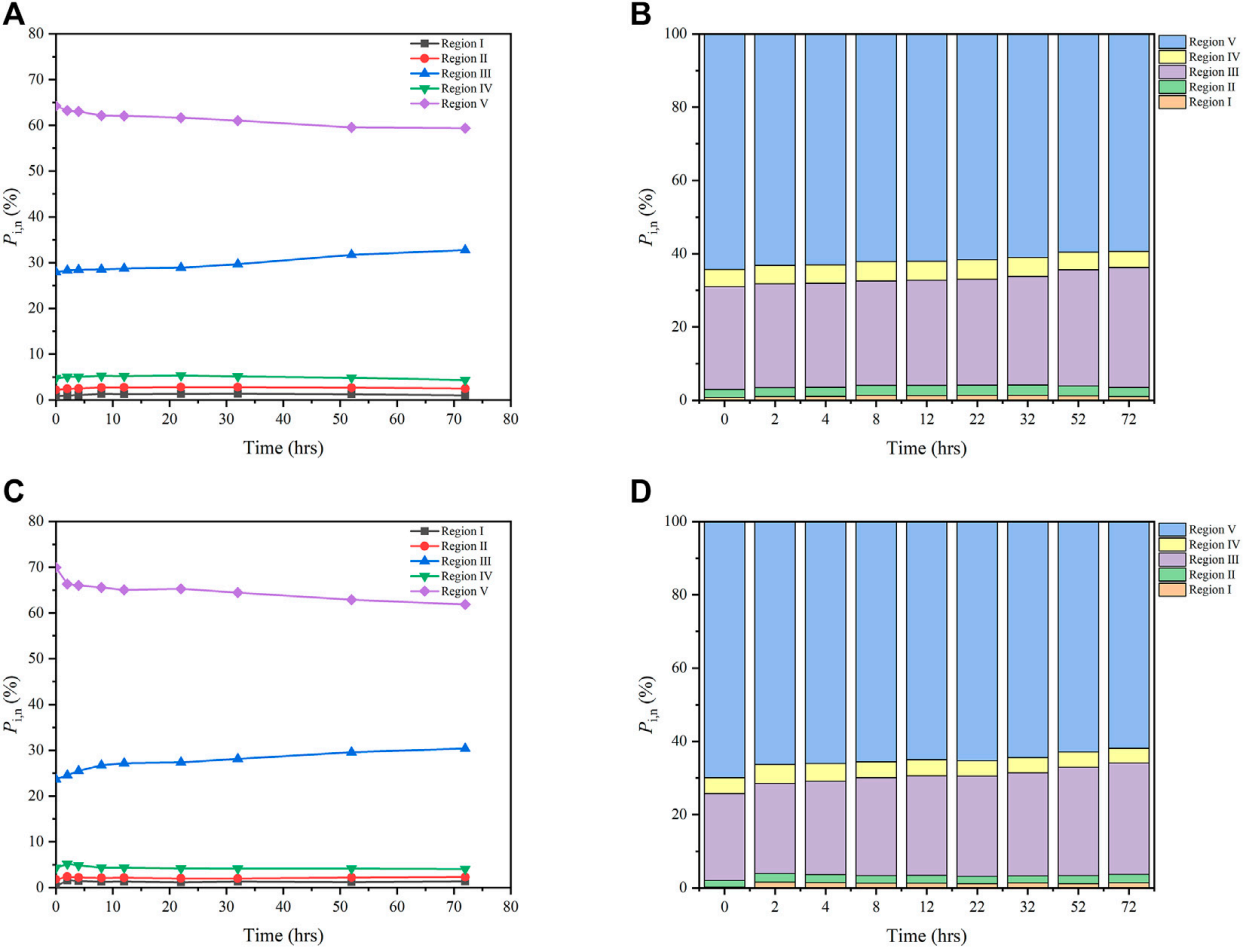
Distributions of *P*_i,n_ of for FA **(A,B)** and HA **(C,D)** as a function of irradiation time, from 0 to 72 h (Region I: tyrosine-like materials; Region II, tryptophan-like materials; Region III, fulvic-like materials; Region IV, soluble microbial byproduct-like materials; Region V, humic-like materials).

Distributions of *P*_i,n_ of both FA and HA with irradiation time are shown in Figure 3. For FA, the values of *P*_I,n_, *P*_II,n_, and *P*_III,n_ increased from 0.81 to 1.02%, from 2.21 to 2.49%, and from 27.97 to 32.76%, respectively, while those of *P*_IV,n,_ and *P*_V,n_ decreased from 4.79 to 4.37% and from 64.24 to 59.37% during 0–72 h of irradiation (Figures 3A,B). For HA, over the same irradiation period, the *P*_I,n_, *P*_II,n_, and *P*_III,n_ values increased from 0.29 to 1.38%, from 1.78 to 2.31%, and from 23.67 to 30.38%, respectively, while *P*_IV,n,_ and *P*_V,n_ respectively decreased from 4.33 to 4.05% and from 69.93 to 61.88% (Figures 3C,D). The maximum increase of *P*_III,n_ related to fulvic-like materials was less than 6.8%, while the maximum decrease of *P*_V,n_ related to humic-like materials was less than 8.1%, for both FA and HA. By contrast, maximal changes in *P*_I,n,_ *P*_II,n_, and *P*_IV,n_ that were related to protein-like materials were less than 0.2–1.1%, for both FA and HA. Although irradiation significantly affected the original EEM of FA and HA, slight changes to the distributions of *P*_i,n_ (<10%) were discernible for both. Such slight variation in the distributions of *P*_i,n_ were also observed in previous studies, related there to the photodegradation of dissolved organic matter in lakes (Zhang et al., 2020). Hence, a PARAFAC analysis was carried out to further investigate the fluorescence properties of FA and HA during irradiation.

### PARAFAC Analysis of HSs

**FIGURE 4:**
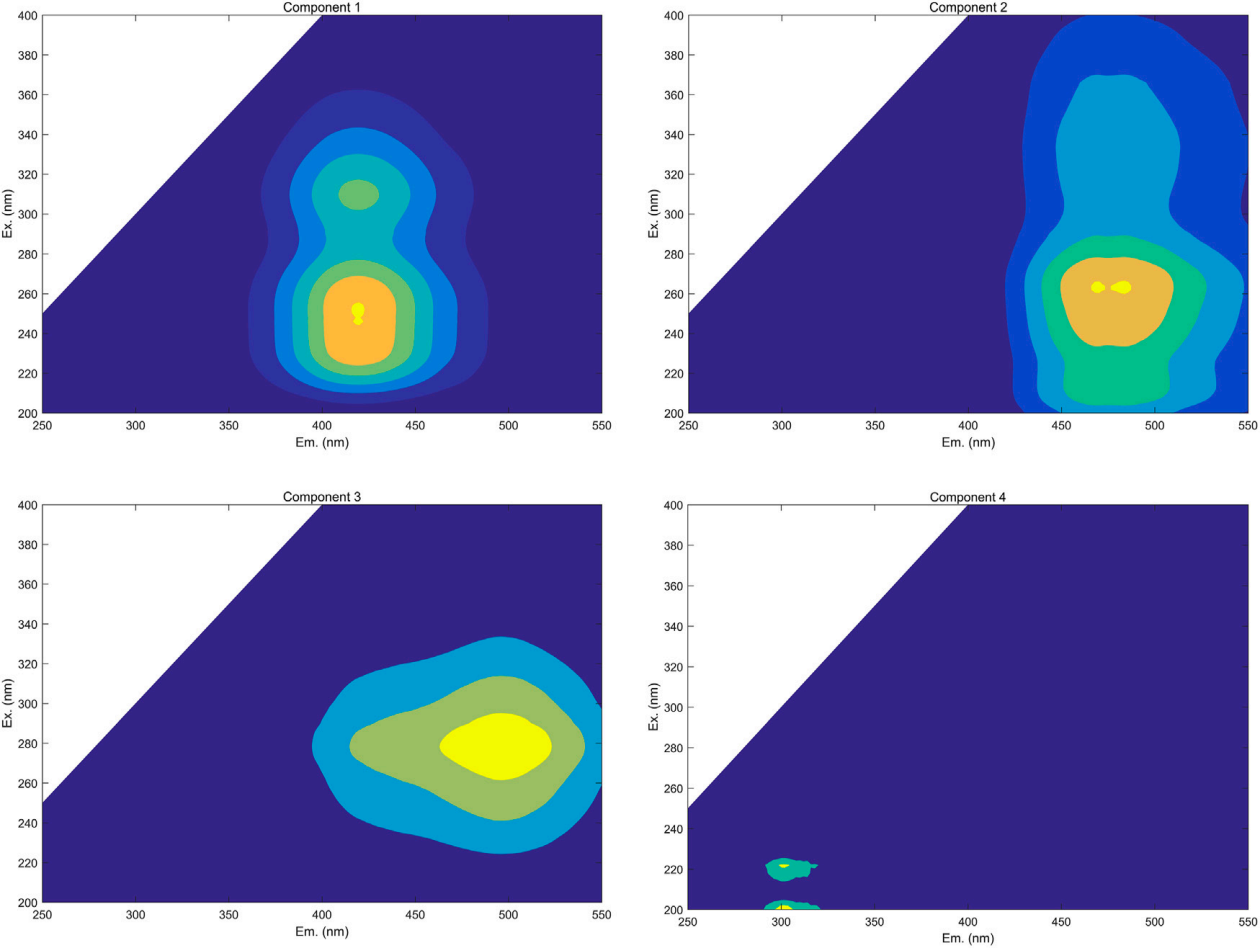
Identified PARAFAC components of FA and HA that were isolated from forest soil (**Component 1**, fulvic-like components composed of both carboxylic-like and phenolic-like chromophores; **Component 2**, terrestrial humic-like components primarily composed of carboxylic-like chromophores; **Component 3**, microbial humic-like components overwhelming composed of phenolic-like fluorophores; **Component 4**, protein-like components).

The spectra of individual components were successfully deconvoluted via the PARAFAC analysis of EEMs of all FA and HA samples during their irradiation. Using the PARAFAC models’ residual analysis and split-half analysis (Maqbool and Hur, 2016; Song et al., 2017), the appropriate number of components was determined to be four (Figure 4).

To identify these four components generated by the PARAFAC analysis, model loadings were uploaded into OpenFluor and compared with the previous research. The Tucker congruence coefficient (TCC) was used to evaluate the consistency of spectra between components derived from different samples and models (Murphy et al., 2011). Multiple strong matches (i.e., TCC >0.95) for all of the components were detected (20, 13, 9, and 3 matches for the components C1, C2, C3, and C4, respectively) in the OpenFluor database. Some information, including the locations of peaks, the maximum intensity of fluorescence, components’ categories, and primary compounds of PARAFAC components are summarized in Table 1. Through their comparison with the OpenFluor database, C1 (Ex/Em: 250–310/420 nm), C2 (Ex/Em: 262/470 nm), C3 (Ex/Em: 280/496 nm), and C4 (Ex/Em: 222/302 nm) were categorized here, respectively, as a fulvic-like component composed of both carboxylic-like and phenolic-like chromophores, a terrestrial humic-like component primarily composed of carboxylic-like chromophores, a microbial humic-like component mostly composed of phenolic-like fluorophores, and a protein-like component (Murphy et al., 2011; Yan et al., 2013; Derrien et al., 2019; Kida et al., 2019). The Em wavelength was longer for C3 than C2, which meant the microbial humic-like components harbored greater aromaticity and a larger scale for the π -conjugated system than did the terrestrial humic-like component (Yu et al., 2020).

**TABLE 1:** Parameters and categories of the individual PARAFAC components for FA and HA during irradiation.

Samples	Components	Ex/Em (nm)	*F*_max_ (a.u.)		α (a.u./hrs)		Degradation percentage	Component categories	Primary compounds
			Before irradiation	After irradiation	0–12 h	12–72 h			
FA	C1	250,310/420	322.23	116.03	3.47	0.37	36.01%	Fulvic-like	Carboxylic-like and phenolic-like
	C2	262/470	103.63	60.46	1.81	0.33	58.34%	Terrestrial humic-like	Carboxylic-like
	C3	280/496	62.68	0.00	4.33	0.80	0%	Microbial humic-like	Phenolic-like
	C4	222/302	17.89	12.69	—	—	—	Protein-like	
HA	C1	250,310/420	100.99	297.69	12.29	0.79	294.77%	Fulvic-like	Carboxylic-like and phenolic-like
	C2	262/470	92.01	217.31	7.21	0.83	236.18%	Terrestrial humic-like	Carboxylic-like
	C3	280/496	233.46	22.48	3.98	0.71	9.63%	Microbial humic-like	Phenolic-like
	C4	222/302	22.54	52.05	—	—	—	Protein-like	

*F*_max_, maximum fluorescence intensity of the PARAFAC components;

*α*, the increase or decrease in fluorescence intensity per hour;

—, data not available.

**FIGURE 5:**
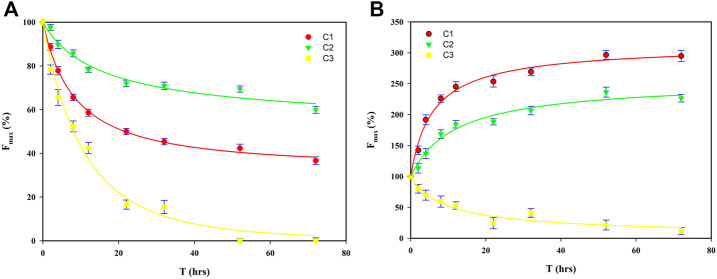
Fitted regressions of *F*_max_ of C1–C3 for FA **(A)** and HA **(B)** for the first-order kinetic model (C1, fulvic-like component; C2, terrestrial humic-like component, C3, microbial humic-like component).

Before irradiation, for FA, the *F*_max_ of C1 (322.23 a.u.) was greater than that of C2 (103.63 a.u.), followed by C3 (62.68 a.u.), and it was least in C4 (17.89 a.u.), while for HA, the *F*_max_ was greatest for C3 (233.46 a.u.), followed by C1 (100.99 a.u.), C2 (92.01 a.u.), and then C4 (22.54 a.u.) (Table 1). The C4 component, however, is not discussed here due to its *F*_max_ being negligible (Supplementary Figure S4; Table 1); this phenomenon was also reported by Yamashita and Jaffe (2008), who instead focused on differences in metal binding among individual PARAFAC components. The initial *F*_max_ values of those components suggested the fulvic-like and humic-like components were the major components for both FA and HA, a result consistent with our analysis of the original EEM using FRI (Figure 2). The variation in the *F*_max_ of components for both FA and HA during the irradiation process is shown in Figure 5. After irradiation for 72 h, for FA, the *F*_max_ of C1–C3 decreased respectively to 36.01, 58.34, and 0%, whereas for HA the *F*_max_ of C1 and C2 increased to 294.77 and 236.18%, respectively, but that of its C3 decreased to 9.63% (Table 1). In comparison with both C1 and C2, a greater decrease of C3 was obtained, indicating a greater change in chemical structure and/or configuration for microbial humic-like components than either the terrestrial humic-like or fulvic-like components during the irradiation of FA. A faster decline in the humic-like than fulvic-like component was also reported by Zhang et al. (2020), who had focused on the irradiation of DOM from artificial reservoirs on a krastic plateau. A greater reduction in the microbial humic-like component than terrestrial humic-like component was also found during an investigation of the influence of pH on DOM’s photodegradation in the Suwannee River (Timko et al., 2015). In our study, the *F*_max_ of C1 and C2 of HA increased, which is inconsistent with its dynamics in FA during the irradiation process (Figure 5). That increased PARAFAC component was also observed for irradiated DOM from a sub-alpine lake (Du et al., 2016), a finding attributable to a greater aromaticity and photorefractive tendency. The fluorescence intensity of each component in FA was significantly decreased under light conditions, which was thought to be related to the direct disruption of the structure of high molecular weight polyaromatic compounds. In the study of photodegradation of aquatic humic substances isolated from Negro river, the irradiation caused the decrease in fluorescence by dissociating conjugates and double bonds and reduced the π electronic density (Rodriguez-Zuniga et al., 2008). The enhanced fluorescence intensity of C1 and C2 in HA can be explained by the breakage of hydrogen bonds Patel-Sorrentino et al. (2004) studied the effect of UV-vis light on the fluorescence of DOM in the Amazon basin, they conclude that the release of H^+^ leads to a more dispersed conformation of the distribution, which promotes the activation of the initially hidden fluorophores in the aggregates. Plausible mechanisms underpinning the increase of both C1 and C2 during the irradiation of HA should be further investigated and discussed in future work. The change ratio (*α*) was defined here as the decrease/increase of fluorescence intensity per hour during the irradiation of HSs. For FA, the *α* of C1–C3 was 1.81–4.33 a.u./h during its irradiation for 0–12 h, and 0.33–0.80 a.u./h when irradiate for 12–72 h (Table 1). For HA, the *α* of C1–C3 had a range of 3.98–12.29 a.u./h during 0–12 h of irradiation, and 0.71–0.83 a.u./hrs under irradiation for 12–72 h (Table 1). This variation inferred from *α* indicated a higher photodegradation rate in the early than late stage of irradiation. An apparent two stage-effect of irradiation was also reported by (Lou and Xie, 2006), who investigated the effects of photodegradation on the molecular weight of DOM. We discuss this phenomenon in the next section.

### Kinetic Characteristics of Photochemical of HSs

Kinetic models are powerful methods to evaluate the photochemical reactivity process (Hur et al., 2011; Zhao et al., 2018). For instance, the photochemical reactivity of HSs derived from leaf litter and the Suwannee River’s fulvic acid were estimated by the first-order kinetic model fitted to fluorescence spectroscopy data (Hur et al., 2011). The photodegradation process of aquatic DOM derived from cyanobacterial blooms has been evaluated by the first-order kinetic model with UV irradiation (Xu and Jiang, 2013). The zero-order and first-order kinetic models were both used to evaluate the photodegradation process of DOM from Lake Hongfeng and Baihua (Zhang et al., 2020). Here, zero-order and first-order kinetic models were also applied to fit the change of *F*_max_ during the entire photochemical reactivity process for FA and HA from forest soil. We used *R* ^*2*^ (the fitting parameter adjusted square of correlation coefficient) to examine the fitness of the kinetic models to the data. Some basic information, including rate constant (*k*), *R* ^2^, *F*_end_, and half-life ($T_{12}$), are summarized in Table 2. In previous research, the photodegradation rates of *F*_max_ of components identified by the PARAFAC analysis matched well with the first-order kinetics for the photodegradation of DOM from urban stormwater runoff (*R* ^2^ = 0.821–0.990) and lake (*R* ^2^ = 0.891–0.988) (Zhao et al., 2018; Zhang et al., 2020). Here, the *R* ^2^ values (*R* ^2^ = 0–0.754) for the fitted zero-order model were smaller than for the first-order one (*R* ^2^ = 0.908–0.990), which indicated the latter model was more suitable to evaluate the photochemical reactivity process (Table 2). Accordingly, the first-order kinetic model was applied to evaluate the photochemical reactivity process of both FA and HA. The *k*_1_ values of C1 (0.105 for FA; 0.154 for HA) were greater than those of C2 (0.059 for FA, 0.079 for HA) and C3 (0.079 for both FA and HA) (Table 2). Notably, the fulvic-like component (C1) displayed faster photochemical reactivity than either the terrestrial humic-like (C2) or microbial humic-like (C3) components, for both FA and HA. Specifically, the microbial humic-like components of FA and HA had a similar photodegradation rate. The photodegradation rates of the fulvic-like and terrestrial humic-like components were 47.04 and 33.62% higher in HA than FA, respectively (Table 2). The *F*_end_ of C1, C2, and C3 were 40.55, 63.64, and 2.76 units for FA, and 276.09, 226.25, and 21.82 units for HA, respectively. The $T_{12}^{1}$ of C1, C2, and C3 respectively were 6.61, 11.77, and 8.73 h for FA, and likewise 4.50, 8.81, and 8.76 h for HA. The range of $T_{12}^{1}$ (4.50–11.77 h) for HSs in our study not unlike that for mangrove-derived DOM in seawater (*T*_1/2_ < 12 h) (Scully et al., 2004), as well as the DOM produced from leaves (*T*_1/2_ = 9 h) (Maie et al., 2008). The $T_{12}^{1}$ parameter results indicated the stability of components varied in the order of terrestrial humic-like component > microbial humic-like component > fulvic component. Extensive research has been conducted on the stability of the components of DOM from terrestrial and aquatic sources. Our results are thus consistent with the stability of components ranked as humic-like > fulvic-like that was reported for the photodegradation of DOM from lakes (Zhang et al., 2020).

**TABLE 2:** Estimated parameters from the zero-order and first-order kinetic simulations of the photochemical reactivity of FA and HA.

Samples	Components	Zero-order kinetic model			First-order kinetic model			
		*k*_0_ (hrs^−1^)	$T_{12}^{0}$ (hrs)	*R* ^2^	*k*_1_ (hrs^−1^)	$T_{12}^{1}$ (hrs)	*F*_end_	*R* ^2^
FA	C1	1.1153	44.83	0.330	0.1048	6.61	40.55	0.990
	C2	0.6269	79.76	0.754	0.0589	11.77	63.64	0.954
	C3	1.8221	27.44	0.426	0.0794	8.73	2.76	0.964
HA	C1	4.2463	22.94	0.000	0.1541	4.50	276.09	0.965
	C2	2.4193	28.14	0.416	0.0787	8.81	226.25	0.956
	C3	1.4881	33.60	0.472	0.0791	8.76	21.82	0.908

*k*_0_ and *k*_1_, the rate constants of the zero-order and first-order kinetic models, respectively.

$T_{12}^{0}$ *and* $T_{12}^{1}$, the half-life values of the zero-order and first-order kinetic models, respectively**

Fend, the final fluorescence intensity caused by irradiation.

## Conclusion

The photochemical reactivity of HSs was investigated by EEM combined with FRI and PARAFAC and kinetic models. Three main peaks of Peak A (Ex/Em: 308–312/422–434 nm), Peak B (Ex/Em: 254–260/430–446 nm), Peak C (Ex/Em: 228–234/424–444 nm) for FA, and another one, Peak D (Ex/Em: 272/484 nm) for HA, were observed in the EEMs before apply the irradiation treatment. The positioning of those peaks underwent a differential red shift during the irradiation process, this related to the changed structural composition of the FA and HA components. Their regional distributions indicated that the humic-like and fulvic-like were the main components of the FA and HA samples. Humic-like materials were distinguished by the greatest changes, followed by the fulvic-like materials and minimal changes of protein-like materials while irradiated. We determined four components of HSs via the PARAFAC analysis, namely a fulvic-like one composed of both carboxylic-like and phenolic-like chromophores (C1), terrestrial humic-like one primarily composed of carboxylic-like chromophores (C2), microbial humic-like one overwhelmingly composed of phenolic-like fluorophores (C3), and protein-like one (C4). During the photochemical reactivity process, the *F*_max_ of C1 and C2 of FA decreased to 36.01 and 58.34%, while that of C3 for FA and HA fell to 0 and 9.63%, respectively; in contrast, the *F*_max_ of C1 and C2 of HA increased to 294.77 and 236.18%, respectively. The increased *F*_max_ of C1 and C2 in HA implied greater aromaticity and photorefractive tendencies. The *R* ^2^ values (*R* ^2^ = 0.908–0.990) for the fitted first-order kinetic model significantly exceeded those of the zero-order kinetic model for the photochemical reactivity process of both FA and HA. According to the obtained parameters *k* and T_1/2_, the stability of the major components (C1–C3) could be ranked as terrestrial humic-like > microbial humic-like > fulvic-like, for both FA and HA.

## Data Availability

The original contributions presented in the study are included in the article/Supplementary Material, further inquiries can be directed to the corresponding authors.
