# Supplementary material for: Photochemical Reactivity of Humic Substances in an Aquatic System Revealed by Excitation-Emission Matrix Fluorescence
Source: Front Chem. 2021 May 28;9:679286. doi: 10.3389/fchem.2021.679286 (PMC8193985; doi:10.3389/fchem.2021.679286)
Supplement: Supplementary file 1 [file DataSheet1.docx]

***Supporting information***

***
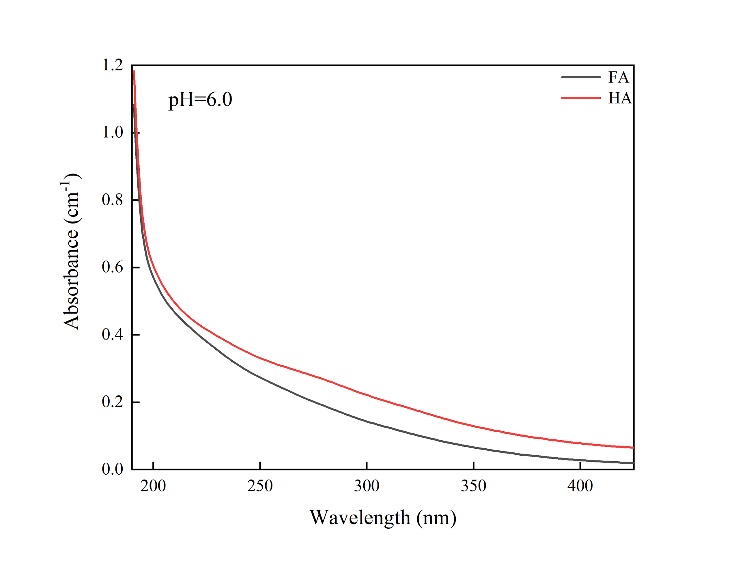
***

Figure S1. The UV-Vis of FA and HA

*
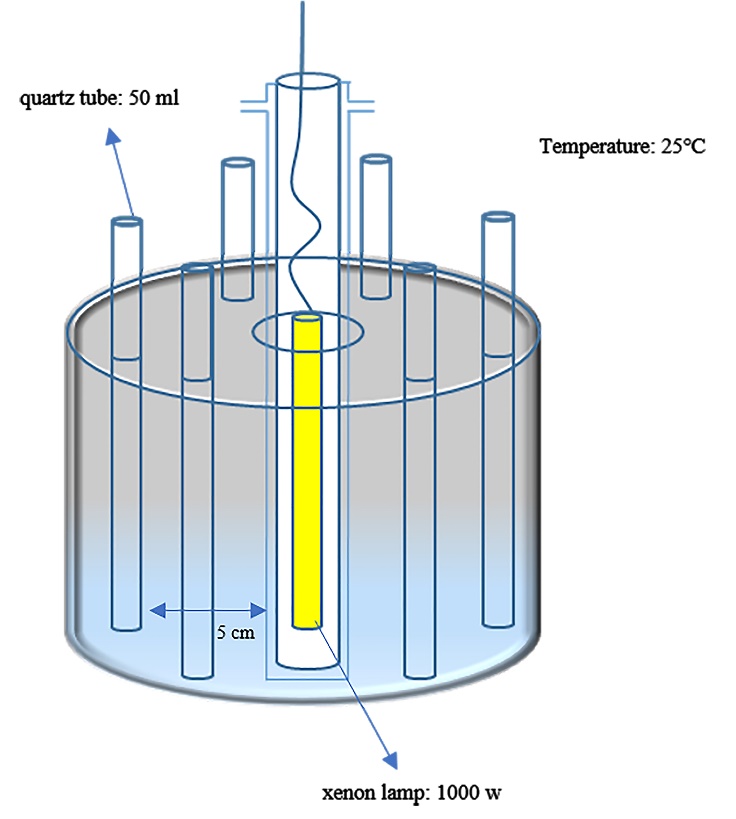
*

Figure S2. The schematic diagram of photochemical reactor

*
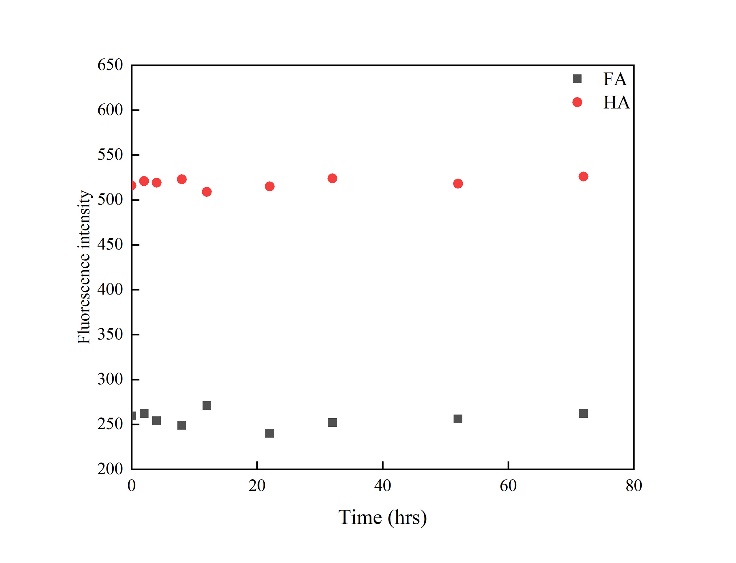
*

Figure S3 The variation of Rayleigh scattered fluorescence intensity of FA and HA with irradiation time


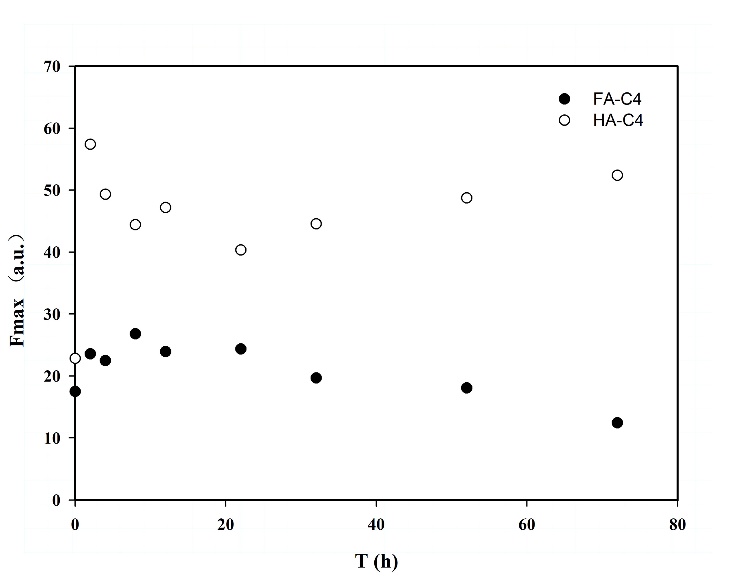


Figure S4. The maximum fluorescence intensity (F_max_) of C4 of FA and HA with irradiation time

Table S1. Elemental compositions and atomic ratios of FA and HA

| Samples | *w* /% | | | | | | Atomic ratios | | |
| --- | --- | --- | --- | --- | --- | --- | --- | --- | --- |
|  | C | H | O | N | S | Ash | H/C | O/C | (N+O)/C |
| FA | 47.17 | 4.66 | 43.84 | 3.33 | 0.61 | 0.48 | 1.18 | 0.7 | 0.76 |
| HA | 57.01 | 5.21 | 30.36 | 3.64 | 0.63 | 2.37 | 1.09 | 0.40 | 0.45 |

Table S2. Distribution of carbon in FA and HA calculated by solid-state ^13^C NMR spectroscopy

| Samples | Distribution of Carbon chemical shift (ppm) (%) | | | | | | | | Aliphatic C ratio (%) |
| --- | --- | --- | --- | --- | --- | --- | --- | --- | --- |
|  | 0-45 | 45-65 | 65-90 | 90-110 | 110-145 | 145-160 | 160-190 | 190-220 |  |
|  | Alkyl C | Methoxyl C | O-alkyl C | Acetal C | Aromatic C | Phenolic C | Carboxylic C | Carbonyl C |  |
| FA | 22.69 | 10.15 | 10.15 | 3.87 | 21.40 | 5.54 | 5.54 | 4.24 | 36 |
| HA | 23.39 | 9.63 | 8.55 | 3.29 | 26.97 | 7.00 | 16.63 | 4.55 | 43 |

Table S3. UV absorbance at 254 and 280 nm of FA and HA

| Samples | SUVA_254_(L/mg C·m) | SUVA_280_(L/mg C·m) |
| --- | --- | --- |
| FA | 6.9 | 5.16 |
| HA | 6.82 | 5.82 |

Table S4. Fluorescence spectral parameters of FA and HA at time 0, 12, and 72 hrs.

| Samples | Time (hrs) | Peak A | | Peak B | | Peak C | | Peak D | |
| --- | --- | --- | --- | --- | --- | --- | --- | --- | --- |
|  |  | Ex/Em | Int.^a^ | Ex/Em | Int.^a^ | Ex/Em | Int.^a^ | Ex/Em | Int.^a^ |
| FA | 0 | 308/428 | 270.9 | 260/432 | 382.5 | 227/424 | 390.3 | - | - |
|  | 12 | 308/430 | 147.9 | 262/434 | 230.1 | 232/430 | 232.7 | - | - |
|  | 72 | 310/433 | 85.52 | - | - | 234/432 | 169.1 | - | - |
| HA | 0 | - | - | - | - | - | - | 272/484 | 340.1 |
|  | 12 | 308/430 | 287.7 | 254/436 | 389.5 | 234/440 | 375.5 | - | - |
|  | 72 | 312/434 | 241.4 | 254/446 | 378 | 237/444 | 434.1 | - | - |

-：data not available; ^a^ int.: fluorescence intensity with arbitrary unit (a.u.).
